# Feature Interaction Dual Self-attention network for sequential recommendation

**DOI:** 10.3389/fnbot.2024.1456192

**Published:** 2024-08-16

**Authors:** Yunfeng Zhu, Shuchun Yao, Xun Sun

**Affiliations:** ^1^Suzhou Industrial Park Institute of Service Outsourcing, Suzhou, China; ^2^School of Computer Engineering, Suzhou Vocational University, Suzhou, China

**Keywords:** sequential recommendation, self-attention, feature interaction, dual self-attention, sequential transition patterns

## Abstract

Combining item feature information helps extract comprehensive sequential patterns, thereby improving the accuracy of sequential recommendations. However, existing methods usually combine features of each item using a vanilla attention mechanism. We argue that such a combination ignores the interactions between features and does not model integrated feature representations. In this study, we propose a novel Feature Interaction Dual Self-attention network (FIDS) model for sequential recommendation, which utilizes dual self-attention to capture both feature interactions and sequential transition patterns. Specifically, we first model the feature interactions for each item to form meaningful higher-order feature representations using a multi-head attention mechanism. Then, we adopt two independent self-attention networks to capture the transition patterns in both the item sequence and the integrated feature sequence, respectively. Moreover, we stack multiple self-attention blocks and add residual connections at each block for all self-attention networks. Finally, we combine the feature-wise and item-wise sequential patterns into a fully connected layer for the next item recommendation. We conduct experiments on two real-world datasets, and our experimental results show that the proposed FIDS method outperforms state-of-the-art recommendation models.

## 1 Introduction

With the development of the Internet, sequential recommendation has been widely used in business scenarios (e.g., e-commerce recommendation, media recommendation, and ad click prediction). In such scenarios, the user's historical behaviors can be organized as a chronological sequence of activities. Moreover, sequential recommendation aims to recommend the next item that the user is likely to interact with in the near future based on the user's historical behaviors.

A large number of methods have been proposed for sequential recommendation. Traditional sequential models are usually based on Markov Chain (MC) (Chen et al., 2015; He and McAuley, 2016). A classic model, Factorizing Personalized Markov Chain (FPMC) (Rendle et al., 2010), has been introduced to factorize user-specific transition matrices over Markov Chain, which assumes that the next action is only related to the previous one. However, with the Markov assumption, an independent combination of the past interactions may limit the performance of recommendation (Xu et al., 2019). Recently, with the success of deep learning, many methods based on Recurrent Neural Network (RNN) have emerged (Hidasi et al., 2016; Zhu et al., 2017). These RNN-based methods usually employ the last hidden state of RNN as the user representation, which is used to predict the next action. Despite the success, RNN models are hard to preserve users' long-term dependencies, even using well-designed cell structures such as Long Short-Term Memory (LSTM) and Gated Recurrent Unit (GRU). Khandelwal et al. (2018) demonstrate that language models using LSTM can apply approximately 200 context tokens on average. However, only 50 nearby tokens can be sharply distinguished, which reveals that even LSTM has trouble in capturing long-range dependencies. In addition, RNN-based methods need to propagate relevant information step by step, which makes it hard to parallelize (Zhang et al., 2019).

More recently, the self-attention mechanism has achieved great success in natural language processing (Vaswani et al., 2017), which also makes outstanding contributions to sequential recommendation. Compared with RNN, self-attention is more suitable for grasping and preserving the long-term dependencies as it allows the model to interact with any step regardless of distance. Kang and McAuley (2018) proposed the Self-Attentive Sequential Recommendation model (SASRec) that applies a self-attention mechanism to replace traditional RNNs for sequential recommendation and achieves remarkable performance. However, SASRec only considers the sequential patterns between items, ignoring the sequential patterns between features, which is incomplete. In actual scenarios, users' behaviors usually also have transition patterns at the item feature level. A very promising idea to solve the problem is to introduce feature-wise into the model to reduce the prediction space to improve recommendation accuracy. Zhang et al. (2019) and its enhanced version Hao et al. (2023) proposed the FDSA model to capture the full sequential patterns from the item-wise and the feature-wise, where a simple vanilla attention operation is used to obtain the integrated feature representation. Though FDSA captures the feature-wise transition patterns and achieves state-of-the-art performance, it generates the feature combinations using the vanilla attention, which assumes that features are independent of each other. This assumption is obviously not realistic (Yun et al., 2019). For instance, women like skirts, while men prefer pants, indicating there are certain dependencies between gender and category. The vanilla attention applied in FDSA(Zhang et al., 2019) and its enhanced version (Hao et al., 2023) is not carefully designed for learning integration features, and it cannot learn effective integrated features. Capturing the dependencies between the features of an item can help learn meaningful and integrated feature representations, and higher-order feature combinations are crucial for good performance (Lian et al., 2018).

In this study, we propose a novel Feature Interaction Dual Self-Attention Network (FIDS) model for sequential recommendation, which utilizes dual self-attention to capture feature interactions and sequential transition patterns. Specifically, we first utilize self-attention to model feature interactions for each item in the sequence, in which each feature is allowed to interact with all other features and is able to automatically identify relevant features to form meaningful higher-order features using a multi-head attention mechanism. Then, we adopt two independent self-attention networks to capture the transition patterns of the item sequence and the integrated feature sequence, respectively. Moreover, we stack multiple self-attention blocks and add residual connections at each block. For self-attention capturing feature interactions, multiple blocks can model interactions at different orders, and residual connections can combine interactions of different orders. For self-attention capturing sequential patterns, stacking multiple blocks can learn more complex item transitions, and residual connections help propagate the visited items' embedding (or integrated features' embedding) to the final layer. Finally, we conduct extensive experiments on two real-world datasets. Our experimental results demonstrate that considering feature interaction can significantly improve the accuracy of the recommendation.

The main contributions of this study are summarized as follows:

- To the best of our knowledge, this is the first study to learn feature interactions and capture sequential patterns all in the unified self-attention mechanism.
- We propose a novel Feature Interaction Dual Self-attention network (FIDS) model for sequential recommendation, which adopts dual self-attention to model the dependencies between items and the dependencies between features, respectively. Specifically, we first utilize self-attention to model the feature interactions for each item to form meaningful higher-order features. Then, we adopt two independent self-attention networks to capture the transition patterns of the item sequence and the integrated feature sequence. Finally, we combine the feature-wise and item-wise sequential patterns to a fully connected layer for the next item recommendation.
- We conduct extensive experiments on two real-world datasets and demonstrate that our proposed method outperforms the state-of-the-art methods.

## 2 Related work

In this section, we discuss related work from two aspects, which are sequential recommendation and attention mechanism.

### 2.1 Sequential recommendation

Most of the existing sequential recommendation methods are concentrated on Markov Chain-based models and neural network-based models. In essence, the first-order Markov Chain captures the transition relationship between the current action and the previous action, while the higher-order Markov Chain assumes that the next action is related to several previous actions. In general, the users' former behavior has a more significant impact on the following action, so the first-order MC-based models can still achieve excellent performance. He et al. (2017) proposed TransRec model, considering the first-order Markov Chain. Rendle et al. (2010) combined Matrix Factorization and Markov Chain to model sequential patterns. He and McAuley (2016) dedicated modeling sequential relationships using higher-order Markov Chains and can make meaningful recommendations even in sparse environments. However, models based on Markov Chains rely on strong assumptions, which may limit the recommendation performance. Recently, with the advancement of deep learning, many neural network-based sequential recommendation methods have emerged. Hidasi et al. (2016) adopted GRU to model transitions between items. Despite its success, RNN-based methods still have problems in maintaining long-term user preferences and parallel processing. Moreover, Lv et al. (2021), Manotumruksa and Yilmaz (2020), and Ren et al. (2020) utilize the generative adversarial network to assist sequential recommendation and improve the model performance by enhancing the generalization of the model. Tolstikhin et al. (2021) hope to capture sequence information using a simple MLP structure which may facilitate the simplification of computation. Recently, numerous studies (Chen et al., 2022; Li et al., 2023; Qin et al., 2023) have suggested employing contrastive learning in sequential recommendation (SR) to enhance user representation. However, these sequential recommenders focus only on item sequences and fail to utilize valuable auxiliary information.

### 2.2 Attention mechanism

In recent years, attention mechanism has been widely used in various tasks, including machine translation (Huang et al., 2016; Miculicich et al., 2018; Zhang J. et al., 2018), computer vision (Jaderberg et al., 2015; Wang et al., 2017; Hu et al., 2018), and recommendation system (Zhang S. et al., 2018). The success of the *Transformer* (Vaswani et al., 2017) and *BERT* (Devlin et al., 2019), which can model syntactic and semantic patterns between words in a sentence very efficiently, stimulates the development of the self-attention mechanism in sequential recommendation. Kang and McAuley (2018) and Sun et al. (2019) employed the self-attention mechanism to model sequential patterns and proved that the self-attention network is superior to RNN/CNN-based models. Zhou et al. (2018) proposed an attention-based user behavior modeling framework, which projects heterogeneous user behaviors into multiple potential semantic spaces, where the influence between behaviors is captured by self-attention. Huang et al. (2018) also captured the polymorphism of user behaviors through a feature-wise self-attention network and dynamically modeled the contextual dependency via the forward and backward position encoding matrices. Lately, Zhang et al. (2019) focused on conducting the feature sequence via vanilla attention and modeling sequence transition patterns from the feature-wise and item-wise.

#### 2.2.1 Difference

The methods mentioned above either only model sequential patterns from a single level (i.e., item-wise) or coarsely integrate feature representations with vanilla attention, which cannot model accurate integrated features and may limit the accuracy of recommendations. Inspired by Song et al. (2019), who adopted a multi-head self-attention to capture feature interactions automatically for Click-Through Rate (CTR) prediction. In this study, we learn feature interactions and capture item-wise and feature-wise sequential patterns under a unified self-attention framework.

## 3 Proposed model

In this section, we introduce the Feature Interaction Dual Self-attention network (FIDS) model. We first formulate the problem definition and then present the architecture and the details of our proposed model.

### 3.1 Problem statement

Sequential recommendation aims to predict the next item that the user interacts with, based on his/her historical interaction sequence. We formulate the sequential recommendation before introducing our proposed model details. We let $U=\left\{u_{1},u_{2},...,u_{\left|U\right|}\right\}$ denote the set of users and $I=\left\{i_{1},i_{2},...,i_{\left|I\right|}\right\}$ represent the set of items, where |U| and |I| represent the number of users and items, respectively. We use $S=\left\{s_{1},s_{2},...,s_{\left|S\right|}\right\}$ to represent the sequence of items that the user has interacted with in a chronological order, where $s_{i}\in I$. In addition, item $s_{i}\in S$ corresponds to a set of features $A_{i}=\left\{{a_{i}}_{1},{a_{i}}_{2},...,{a_{i}}_{m}\right\}$, where *m* represents the number of features of each item in the dataset. The goal is to recommend the next item i∈I that user u∈U might interact with. For clarity, Table 1 lists the symbols involved and their definitions.

**Table 1:** Table of notations.

**Notation**	**Description**
U, I, C	Set of users, items and features
|U|, |I|, |C|	The number of users, items and features
S	Item sequence of user history interaction
$A_{*}$	Sets of features of an item
*n*	Maximum sequence length
*m*	Number of features of one item
*n*_*h*_	Number of self-attention heads
*b*	Number of self-attention blocks
*d*	Latent vector dimension
$\text{U}\in {\mathbb{R}}^{|U|\times d}$	User embedding matrix
$\text{I}\in {\mathbb{R}}^{|I|\times d}$	Item embedding matrix
**P**∈ℝ^*n*×*d*^	Position matrix
**S**∈ℝ^*n*×*d*^	Input item embedding matrix
${\text{A}}_{*}\in {\mathbb{R}}^{m\times d}$	Input feature latent matrice of an item
E^*^∈ℝ^*m*×*d*^	Output of self-attention in automatic feature interaction layer
	**F**∈ℝ^*n*×*d*^	Integrated feature sequence matrix
${\text{O}}_{\text{f}}^{\left(b\right)}\in {\mathbb{R}}^{n\times d}$	Feature embeddings after the feature-wise self-attention layer
	${\text{O}}_{\text{s}}^{\left(b\right)}\in {\mathbb{R}}^{n\times d}$	Item embeddings after the item-wise self-attention layer
*L*		The objective function

### 3.2 The architecture of FIDS

We propose a novel Feature Interaction Dual Self-Attention Network, the basic idea of adopting a dual self-attention network to generate an accurate feature sequence by considering feature interactions and capturing the full sequential patterns from item-wise and feature-wise. We mainly consider the following characteristics of users' sequential behaviors.

1) The users' sequential behavior is not only related to the item sequence but also closely related to the feature-wise sequential pattern.
2) For each item, feature interaction can capture a more comprehensive integrated feature, thereby enhancing the expressive ability of feature-wise modeling sequential dependencies.

#### 3.2.1 Automatic feature interaction

Modeling feature interactions with the self-attention mechanism has proven effective in click-through rate (CTR) prediction tasks (Song et al., 2019; Yun et al., 2019). Inspired by them, we use *n* self-attention modules to model the interaction between the features corresponding to *n* items automatically in the automatic feature interaction layer, where *n* represents the historical interaction number of the input sequence. Each self-attention module acts on one item's features and generates integrated higher-level features. Then, we use the vanilla attention to select and merge its output into a *d*-dimensional feature vector for each item. In this way, meaningful feature representations have been generated. The second problem mentioned above has been solved.

#### 3.2.2 Capturing transition patterns

Zhang et al. (2019) proved that only the item level is not enough to model the entire sequence pattern. Here, we model the feature-wise transition patterns and the item-wise transition patterns in the feature-wise self-attention layer and the item-wise self-attention layer, respectively. More specifically, we use two self-attention networks with independent parameters to model item-wise and feature-wise transition patterns.

**Figure 1:**
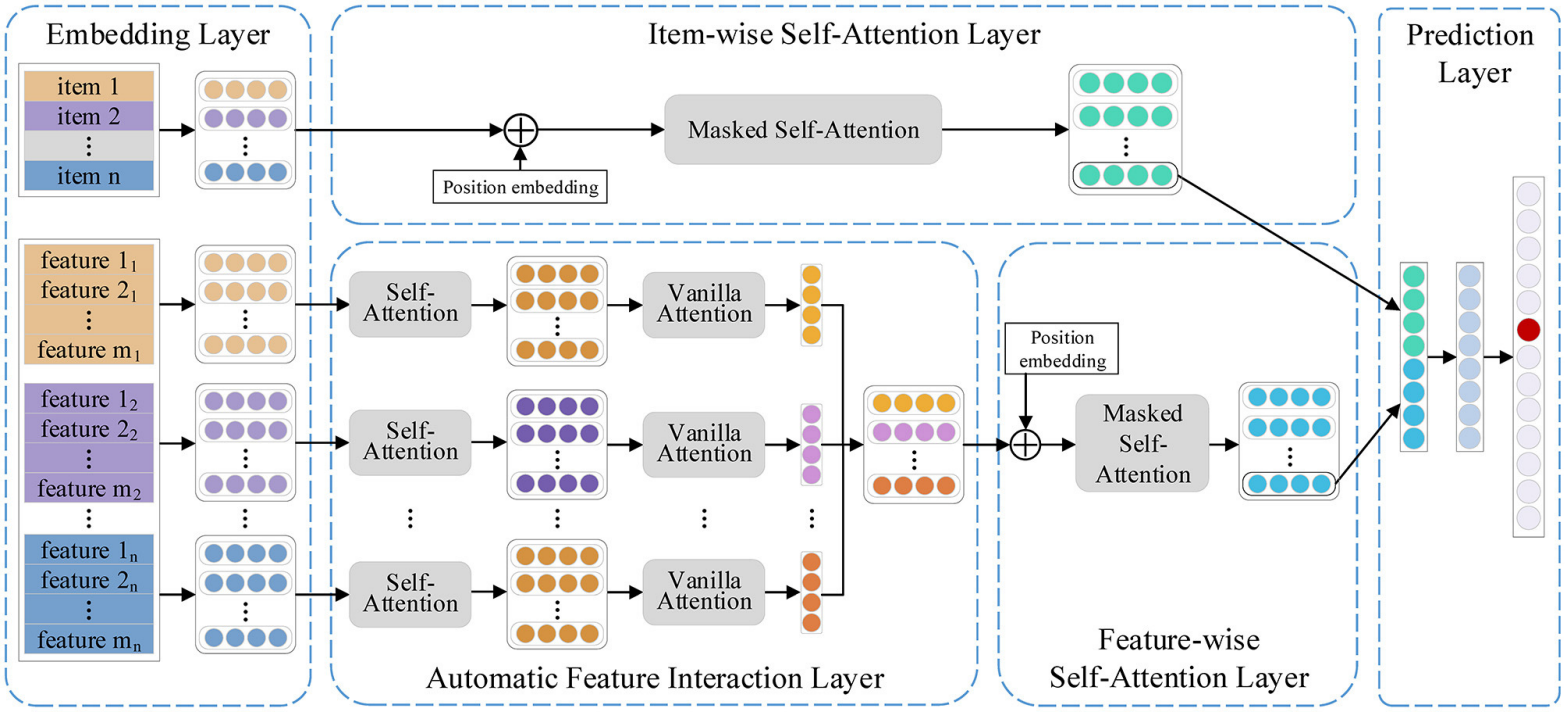
Framework of feature interaction dual self-attention network.

As shown in Figure 1, FIDS consists of five parts, namely, an embedding layer, an automatic feature interaction layer, an item-wise self-attention layer, a feature-wise self-attention layer, and a prediction layer. Specifically, we first project the items and relevant features into dense vector representations. Then, the automatic feature interaction layer adopts multi-head self-attention networks to learn higher-order interactions between features automatically and generate the feature sequence. Subsequently, the feature-wise sequential patterns and the item-wise sequential patterns are learned in the feature-wise self-attention layer and the item-wise self-attention layer, respectively. Finally, we combine the two sequential patterns and recommend the next item in the prediction layer. Following, we elaborate on the details of our proposed model FIDS.

### 3.3 Embedding layer

We convert the user historical interaction sequence into a fixed-length sequence *s* = (*s*_1_, *s*_2_, ..., *s*_*n*_), where *n* represents the maximum length that the model can accommodate. If the sequence length is longer than *n*, we intercept the *n* items that the user has recently interacted with. For the length of sequences less than *n*, we adopt a zero-padding strategy. We first map the item sequence into a dense latent matrix **V**∈ℝ^*n*×*d*^, where *d* represents the latent dimension. Since the self-attention mechanism does not have position awareness, we generate a learnable position matrix **P**∈ℝ^*n*×*d*^ to model the position relationship (Kang and McAuley, 2018). Each item in the sequence corresponds to a set of features, and we generate a feature matrix ${\text{A}}_{i}\in {\mathbb{R}}^{m\times d}$ for item *s*_*i*_, where *m* is the number of features of each item. Then, the original feature sequence can be expressed as a matrix sequence *f* = (**A**_1_, **A**_2_, ..., **A**_*n*_).

In short, the embedding layer generates three sequences: item sequence, position sequence, and feature sequence. We use **S**∈ℝ^*n*×*d*^ and **P**∈ℝ^*n*×*d*^ to represent the item and position sequence embedding matrix respectively. ${\text{A}}_{*}\in {\mathbb{R}}^{m\times d}$ is used to represent an element of the feature sequence.

### 3.4 Automatic feature interaction layer

The critical task at the automatic feature interaction layer is to learn meaningful higher-order combined features. Song et al. (2019) proved that self-attention network can effectively construct higher-order feature interactions in CTR prediction tasks. Inspired by it, once the feature matrix **A**_*i*_ about the *i*-th item is obtained, we use a self-attention mechanism to learn higher-order interactions between features. We adopt the widely used scaled dot-product attention (Vaswani et al., 2017), which is defined as follows:


(1) $$Attention\left(\text{Q},\text{K},\text{V}\right)=softmax\left(\frac{\text{Q}{\text{K}}^{T}}{\sqrt{d}}\right)V$$

where **Q**, **K**, and **V** represent queries, keys, and values, respectively. The term $\frac{1}{\sqrt{d}}$ constrains the scale of the dot products, where *d* is the latent dimension. For the task of learning the higher-order interactions between features, **Q**, **K**, and **V** are all generated by **A**_*i*_. We first transform the feature matrix **A**_*i*_ into three matrices via linear transformation and feed them into *Attention* to learn higher-order interaction features.


(2) $${\text{H}}_{{\text{A}}_{i}}=Attention\left({\text{A}}_{i}{\text{W}}^{Q},{\text{A}}_{i}{\text{W}}^{K},{\text{A}}_{i}{\text{W}}^{V}\right),$$

where **W**^*Q*^, **W**^*K*^, **W**^*V*^∈ℝ^*d*×*d*^ are learnable weights. By doing this, each feature vector is obtained by summing all feature vectors with all attention scores.

#### 3.4.1 Multi-head self-attention

We adopt a multi-head self-attention to map different feature interactions to multiple subspaces and concatenate the outputs of different subspaces:


(3) $$\begin{array}{l} M_{A_{i}}=[h_{1};h_{2};...;h_{n_{h}}]W^{A_{i}},\\ h_{j}=Attention(A_{i}W_{j}^{Q},A_{i}W_{j}^{K},A_{i}W_{j}^{V}), \end{array}$$

where *n*_*h*_ denotes the number of heads in the automatic feature interaction layer. And ${\text{W}}_{j}^{Q}$, ${\text{W}}_{j}^{K}$, ${\text{W}}_{j}^{V}$, and ${\text{W}}^{A_{i}}$ are weight matrixes.

#### 3.4.2 Residual connection

To a certain extent, the deeper the network is, the stronger the expression ability and the better the performance will be. However, the increase of network depth also brings many problems, such as gradient disappearance and gradient explosion. Therefore, simply adding more layers does not directly correspond to better performance. He et al. (2016) proposed residual networks which help propagate lower features to higher features. To preserve the combined features learned previously, we apply residual connections to combine different order features:


(4) $$\begin{array}{cc} {\text{M}}_{{\text{A}}_{i}}^{\prime } & =LayerNorm\left({\text{M}}_{{\text{A}}_{i}}+{\text{A}}_{i}\right), \end{array}$$

where *LayerNorm* is Layer Normalization (Ba et al., 2016), which is used to constrain the parameter range in order to alleviate overfitting, and what we adopt is the same as Kang and McAuley (2018):


(5) $$LayerNorm\left(\text{x}\right)=\alpha ⊙\frac{\text{x}-\mu }{\sqrt{\sigma^{2}+\epsilon }+\beta },$$

where **x** is the assumed input and μ, σ^2^ are mean and variance. ⊙ is the Hadamard product. And α, β are learnable parameters.

#### 3.4.3 Feed-forward network

Although the self-attention network has strong learning capabilities, it still cannot get rid of the fact that it is a linear model. To endow the model with non-linear capabilities and consider the interaction at the dimensional level at the same time, we then add two fully connected layers:


(6) $$O_{A_{i}}=ReLU((M_{A_{i}}^{'}W_{1}+b_{1})W_{2}+b_{2}),$$

where **W**_1_, ${\text{W}}_{2}\in {\mathbb{R}}^{d\times d}$, **b**_1_, ${\text{b}}_{1}\in {\mathbb{R}}^{d}$ are weight matrixes and bias, respectively. In essence, each feature of **O**_**A**_*i*__ has merged the two-order influence of other features on itself.

#### 3.4.4 Multiple self-attention blocks

To capture higher-order combined features, we stack multiple self-attention blocks. We use **SAttB** (Self-Attention Block) to represent the above self-attention process for simplifying; then, the entire process of stacking multiple self-attention blocks can be expressed as


(7) $$\begin{array}{l} \;O_{A_{i}}^{\left(1\right)}=SAttB(A_{i}),\\ \;O_{A_{i}}^{\left(2\right)}=SAttB(O_{A_{i}}^{\left(1\right)}),\\ \;......\\ E^{i}=O_{A_{i}}^{\left(b\right)}=SAttB(O_{A_{i}}^{\left(b-1\right)}), \end{array}$$

where ${\text{O}}_{{\text{A}}_{i}}^{\left(b\right)}\in {\mathbb{R}}^{m\times d}$ is the output after stacking *b* self-attention blocks about item *i*, and *b* (*b*> = 1) is the number of self-attention blocks.

#### 3.4.5 Vanilla attention

Next, we use vanilla attention to merge mixed feature matrix to a feature vector and select which features determine the user's choice:


(8) $$\begin{array}{cc} {\text{f}}_{i} & =\overset{m}{\underset{j}{\sum }}\alpha_{j}{\text{e}}_{j}^{i},\\ \alpha_{j} & =\frac{exp\left({\text{e}}_{j}^{i}\right)}{\sum_{k}^{m}exp\left({\text{e}}_{k}^{i}\right)}, \end{array}$$

where ${\text{e}}_{j}^{i}$ is the *j*-th row of **E**^*i*^. The term ${\text{f}}_{i}\in {\mathbb{R}}^{d}$ is higher-order integrated feature of item *i*. Then, the feature-wise sequence can be translated to *F* = (**f**_1_, **f**_2_, ...**f**_*n*_), where **f**_*i*_ represents the fused high-order feature corresponding to item *i*. And we let **F**∈ℝ^*n*×*d*^ denote the integrated feature sequence matrix.

### 3.5 Feature-wise self-attention layer

Once the feature sequence *F* = (**f**_1_, **f**_2_, ...**f**_*n*_) is obtained, we continue to use a same self-attention network to preserve the contextual information and learn the dependencies between features, and then, we try to generate a transition sequence $F^{\prime }=\left({\text{f}}_{2},{\text{f}}_{3},...{\text{f}}_{n+1}\right)$. The last row of the output matrix in this layer corresponds to the fusion feature of the next item that the user may be interested in.

#### 3.5.1 Position-coding

Since the self-attention network ignores the positional relationship, we add position-coding **P**∈ℝ^*n*×*d*^ to the feature sequence matrix **F** to preserve the order of user interactions:


(9) $$F=\left[\begin{array}{c} f_{1}+p_{1}\\ f_{2}+p_{2}\\ \cdot \cdot \cdot \\ f_{n}+p_{n} \end{array}\right].$$

Then, we send the sum matrix to the self-attention blocks to capture the user's sequential patterns from the feature-wise, which is shown as follows:


(10) $$\begin{array}{l} O_{f}^{\left(1\right)}=SAttB(F),\\ O_{f}^{\left(2\right)}=SAttB(O_{f}^{\left(1\right)}),\\ \;......\\ O_{f}^{\left(b\right)}=SAttB(O_{f}^{\left(b-1\right)}), \end{array}$$

where ${\text{O}}_{\text{f}}^{\left(b\right)}\in {\mathbb{R}}^{n\times d}$ is the learned feature transition matrix, the last row of which can be interpreted as the next fusion feature that the user might be interested in.

#### 3.5.2 Mask

Unlike learning high-level feature interactions, when modeling sequential transition patterns, we must limit the influence of items purchased in future on items purchased in the past due to the inherent sequence of sequences. More specifically, we adjust the attention weights to 0 to eliminate the influence of **f**^*i*^ on **f**^*j*^, where *i*>*j*.

#### 3.5.3 Difference

The automatic feature interaction layer and the feature-wise self-attention layer (or the item-wise self-attention layer, which will be introduced in detail later) are different when using self-attention, although both utilize the attention mechanism. 1) We do not need to consider position-coding when automatically capturing feature interactions as there is no positional relationship between features of an item. However, modeling sequential patterns requires position-coding to learn the location contact. 2) When modeling feature-wise sequential patterns (or item-wise sequential patterns), the impact of future features (or items) on past features (or items) needs to be masked, but no mask is required when capturing feature interactions as there is no order between features. In addition, they have diverse interpretations when using self-attention. Multiple block stacking is used to model different order interactions and learn more complex sequential patterns in the modeling feature interaction task and capturing sequence mode, respectively. Residual connections can combine interactions of different orders in the feature interaction task. When modeling the transition patterns, it helps propagate integrated features' embedding (or the visited items' embedding) to the following layer.

### 3.6 Item-wise self-attention layer

The item-wise self-attention layer aims to learn the dependencies between items. Similar to feature-wise, for a given item sequence *S* = (*s*_1_, *s*_2_, ..., *s*_*n*_), this layer try to learn a transition sequence *S* = (*s*_2_, *s*_3_, ..., *s*_*n*+1_). In detail, we first attach a position-coding to the item sequence **S**. Then, put it into stacked self-attention blocks, as shown follows:


(11) $$S=\left[\begin{array}{c} s_{1}+p_{1}\\ s_{2}+p_{2}\\ \cdot \cdot \cdot \\ s_{n}+p_{n} \end{array}\right],$$

(12) $$\begin{array}{l} O_{s}^{\left(1\right)}=SAttB(S),\\ O_{s}^{\left(2\right)}=SAttB(O_{s}^{\left(1\right)}),\\ \;......\\ O_{s}^{\left(b\right)}=SAttB(O_{s}^{\left(b-1\right)}), \end{array}$$

where the output ${\text{O}}_{\text{s}}^{\left(b\right)}\in {\mathbb{R}}^{n\times d}$ of the last self-attention block is the learned sequential pattern of item-wise. Note that the “Mask” operation is also selected in the item-wise self-attention layer as in the real scene, people do not know what they will purchase in future when they buy items.

### 3.7 Prediction layer

To comprehensively consider feature-wise and item-wise transition patterns, we concatenate the output of the two self-attention layer ${\text{O}}_{\text{f}}^{\left(b\right)}$ and ${\text{O}}_{\text{s}}^{\left(b\right)}$ and then map it to a fully connected layer:


(13) $${\text{Z}}^{u}=\left[{\text{O}}_{\text{f}}^{\left(b\right)};{\text{O}}_{\text{s}}^{\left(b\right)}\right]{\text{W}}_{\text{z}}+{\text{b}}_{\text{z}},$$

where ${\text{W}}_{\text{z}}\in {\mathbb{R}}^{2d\times d}$, ${\text{b}}_{\text{z}}\in {\mathbb{R}}^{d}$ denote the weight matrix and bias, respectively. Finally, given a user *u*, the relevant score of candidate item i∈I is calculated as follows:


(14) $$y_{i,t}^{u}={\text{z}}_{t}^{u}{\text{v}}_{i}^{T},$$

where ${\text{z}}_{t}^{u}$ denotes the *t*-th line of **Z**_*u*_ (*t*∈[1, *n*]), **v**_*i*_ is one of the candidate item embedding, and the *v*_*i*_ is generated based solely on the item ID. We extract the last step to calculate the score in the prediction. We use the product to calculate the score of each candidate item. Then, we sort the scores of all candidate items. The higher the score, the more likely it is the next interactive item of the user.

### 3.8 Training

In training, we randomly sample 100 negative items for each training sequence and minimize the loss function below:


(15) $$L=-\sum_{i\in s}\sum_{t\in [1,n]}[log(\sigma (y_{i,t}))+\sum_{j\notin s}log(1-\sigma (y_{j,t}))].$$

#### 3.8.1 Optimizer

We use the Adam optimizer (Kingma and Ba, 2015) to optimize the network, which designs independent adaptive learning rates for different parameters by calculating the first-order moment estimation and the second-order moment estimation of the gradient. During the evaluation phase, the number of candidate items considered for each user is all items in the dataset. This approach ensures a comprehensive evaluation of the recommendation system's performance.

#### 3.8.2 Dropout

Overfitting is a common problem in neural network learning. Dropout means that during the training of the deep learning network, the neural network unit is temporarily dropped from the network according to a certain probability, and it is shown to be an effective means to alleviate overfitting in various neural networks (Hinton et al., 2012; Krizhevsky et al., 2012; Srivastava et al., 2014; Bouthillier et al., 2016; Volkovs et al., 2017). We also adopt a dropout layer on the input item embedding, the fully connected layer, and the output of the “Mask” operation.

## 4 Experiments

In this section, we first introduce the datasets, baseline methods, evaluation metrics, and parameter settings in our experiments. Then, we compare FIDS with the state-of-the-art baseline methods, presenting experimental results and analyzing the reasons.

### 4.1 Datasets

To compare the performance, we conduct experiments on two real-world datasets: Tmall and MovieLens^1^. Tmall is a comprehensive shopping website. The Tmall dataset is obtained from IJCAI 2015 competition^2^. We filter out users with less than 15 clicks and items with less than 30 clicks by users (Kang and McAuley, 2018). Each item contains three features (i.e., category, seller, and brand). MovieLens is a collection of movie ratings, including seven contextual features in total (i.e., rating, gender, age, occupation, zip-code, year, and genre), where we treat rating as a feature, and we treat a user's features as the items' features that he/she has interacted with for not to waste information. Furthermore, for an item sequence *s* = (*s*_1_, *s*_2_, ..., *s*_*n*_), we use *s* = (*s*_1_, *s*_2_, ..., *s*_*n*−1_) for training and *s*_*n*_ for testing. The feature sequence is treated similarly. Table 2 shows the statistics of the datasets.

**Table 2:** Statistics of datasets.

**Datasets**	**Tmall**	**MovieLens**
#clicks	276,117	1,000,210
#users	16,257	6,040
#items	18,678	3,706
#features of a record	3	7
Avg. #length of a user	15.98	163.82
sparsity	99.91%	95.53%

### 4.2 Baseline methods

We compared our proposed method FIDS with the following competitive models.

**BPR-MF** (Rendle et al., 2009) is based on Bayesian theory to maximize the posterior probability under a priori knowledge, which uses a pairwise ranking loss to optimize the model and combines matrix factorization for recommendation.

**FPMC** (Rendle et al., 2010) is mainly used to predict the likelihood that unknown items will arouse user interest and use this to list item recommendation lists, which combines matrix factorization and Markov Chain for next-basket recommendation.

**GRU4Rec** (Hidasi et al., 2016) employs Gated Recurrent Unit (GRU) to model user sequential behaviors for session-based recommendations. Here, we treat an entire sequence as a session during training.

**TransRec** (He et al., 2017) establishes a third-order relationship between a user, a previously consumed item, and the next item. Furthermore, it embeds the item as a point in the “translation” space, and the user's sequence behavior exists as a translation vector in the space and then predicts the next item that may have behavior through distance calculation.

**Caser** (Tang and Wang, 2018) is proposed for top-N sequential recommendation by modeling recent interacted actions as an “image” and learning sequential patterns via convolution filters.

**SASRec** (Kang and McAuley, 2018) applies a self-attention mechanism for the next item recommendation, which enables it to make predictions based on relatively few actions.

**SASRec+** (Kang and McAuley, 2018) is our extension to the SASRec method involves concatenating item vector representations with category vector representations to serve as the input for the item-level self-attention network.

**MFGAN** (Ren et al., 2020) employs the adversarial generation network to sequential recommendation, which uses a multi-discriminator structure to disentangle different factors to model contextual information and improve the performance of sequential recommendation.

**MLP-Mixer+** (Tolstikhin et al., 2021) is our extended version of the MLP-Mixer model, designed to adapt to sequential recommendation tasks by incorporating explicit item features.

**FDSA** (Zhang et al., 2019) adopts item sequences and feature sequences to model dependencies between items and dependencies between features, respectively.

### 4.3 Experimental setup

#### 4.3.1 Evaluation metrics

To evaluate the performance, we use two general evaluation metrics, that is, Hit Rate (HR@K) and Normalized Discounted Cumulative Gain (NDCG@K). The former evaluates the unordered list of recommendations, and the latter evaluates the ordered sequence. Here, we adopt *K* = {5, 10} for sequential recommendation.

#### 4.3.2 Parameter settings

For the parameters of baselines, we follow the best settings in their studies. In our study, we set the maximum length to 100 in Tmall and the MovieLens dataset to 400. Moreover, the maximum length is also set in the same way in the model SASRec (Kang and McAuley, 2018) and FDSA (Zhang et al., 2019). The learning rate of Tmall and MovieLens is set to 0.0001 and 0.0002, respectively. The number of blocks of all self-attention networks is set to 2 and 3 on Tmall and MovieLens, respectively. For the parameter of the number of heads, we divide all self-attention networks into two categories, used to model feature interactions and sequence transition patterns. On the Tmall dataset, the number of heads of these two types of self-attention is set to 1. For MovieLens, the number of heads of self-attention used to model the sequence transition patterns is set to 4, and for modeling feature interaction, the value is set to 2. The dropout rate is 0.3 in Tmall and 0.2 in MovieLens. The embedding size is set to 128 and 256 on the Tmall and MovieLens datasets, respectively. For all models, the candidate set for evaluation includes one hundred negative examples sampled randomly and one positive example.

### 4.4 Results and discussion

To prove the effectiveness of our proposed model FIDS, we compared it with seven state-of-the-art methods on Tmall and MovieLens. The experimental results are shown in Table 3, and we have the following observations:

**Table 3:** Comparison of model performance on Tmall and MovieLens.

**Datasets**	**Tmall**				**MovieLens**			
**Measures**	**HR@5**	**NDCG@5**	**HR@10**	**NDCG@10**	**HR@5**	**NDCG@5**	**HR@10**	**NDCG@10**
BPR-MF	0.2226	0.1474	0.3255	0.1781	0.3227	0.2033	0.4909	0.2557
FPMC	0.2855	0.2043	0.3944	0.2394	0.3435	0.2194	0.5276	0.2785
GRU4Rec	0.2155	0.1185	0.3082	0.1468	0.3714	0.1900	0.5093	0.2322
TransRec	0.2644	0.1820	0.3772	0.2169	0.3944	0.2548	0.5546	0.3048
Caser	0.3254	0.2337	0.4409	0.2708	0.6289	0.4687	0.7652	0.5131
SASRec	0.3679	0.2679	0.4852	0.3057	0.6753	0.5082	0.7917	0.5462
SASRec+	0.3427	0.2415	0.4714	0.2829	0.6774	0.5192	0.7986	0.5532
MFGAN	0.3889	0.2741	0.4963	0.3231	0.6908	0.5372	0.8090	0.5773
MLP-Mixer+	0.3930	0.2869	0.4059	0.3229	0.7301	0.5602	0.8207	0.5902
FDSA	0.3999	0.2914	0.5122	0.3309	0.7315	0.5642	0.8285	0.5957
FIDS	**0.4360**	**0.3166**	**0.5493**	**0.3533**	**0.7469**	**0.5872**	**0.8376**	**0.6167**
Improv.	9.02%	8.65%	7.24%	6.77%	2.11%	4.08%	1.10%	3.53%

The best score in each column is bolded, while the second-best score is underlined.

First, we can observe that BPR, which does not consider the sequence of user behaviors, performs worse than most sequential-based models (e.g., FPMC and TransRec). This indicates that modeling users' sequential behaviors can enhance the accuracy of recommendations. However, GRU4Rec performs poorly. We analyze that the poor performance of GRU4Rec is caused by the problem of disappearing gradients when RNN captures long-term preferences, so it is hard to model users' long-term preferences. GRU4Rec is more suitable for session-based recommendation. Similarly, Caser employs a convolutional module to combine sequential tokens, organizing them into a matrix format. Caser typically exhibits performance comparable to GRU4Rec.

Second, methods based on the self-attention mechanism, that is, SASRec, MFGAN, FDSA, and FIDS, are superior to other methods, which proves the effectiveness of self-attention in modeling user sequential preferences. Compared with RNN-based and CNN-based models, the advantage of self-attention is that the hidden state obtained at each step contains the information about the entire sequence. SASRec+ outperforms SASRec on the MovieLens dataset but underperforms on the Tmall dataset. This can be attributed to the instability in modeling sequential patterns when concatenating item representations with item feature representations as input vectors for the self-attention mechanism. In essence, self-attention can model the dependencies between an item and all step items, which is the strength of the self-attention inherent structure. In addition, both FDSA and FIDS consider features and exceed SASRec, MLP-Mixer+ and MFGAN, which proves that capturing the dependencies between items alone cannot adequately model the users' sequential behaviors, and the feature sequence also exposes the users' sequential behaviors to some extent.

Finally, compared to FDSA, our proposed model FIDS adaptively learns the features of higher-order interactions via multiple self-attention blocks with residual connection and integrates them with vanilla attention to enhance the representation of elements in feature sequences. From Table 3, we can observe that FIDS exceeds the strongest baseline FDSA by an average of 7.92% and 2.71% on Tmall and MovieLens, respectively. The results prove that considering feature-wise feature interactions can accurately and comprehensively model integrated features. Moreover, our approach outperforms all state-of-the-art methods. This illustrates that FIDS is an effective method for sequential recommendation.

## 5 Ablation analysis

In this section, we construct detailed experiments to analyze two problems: (1) The impact of only item-wise or only feature-wise modeling. (2) Whether feature interaction can positively help model performance.

**(1) The impact of only item-wise or only feature-wise modeling**. We discuss the insufficient of considering only a single sequence pattern by constructing two sub-experiments. We use FIDS-item [identical as the SASRec model essentially (Kang and McAuley, 2018)] to represent a model that only considers item sequences and FIDS-fea to represent a model that only considers feature sequences.

As shown in Table 4, the performance of the FIDS model is 16.48% and 11.21% higher than FIDS-item on average on the Tmall and MovieLens datasets. Comparing FIDS and FIDS-fea, FIDS has an average increase of 1.19% and 0.74% on the two datasets. These increments show that it is necessary to learn sequential transition patterns from item-wise and feature-wise at the same time. In addition, we can observe that the performance of FIDS-fea is better than that of FIDS-item. The reason we analyze is the contribution of introducing features. Introducing contextual features can alleviate the problem of sparse data to a certain extent. Moreover, comparing the two datasets, the improvement of introducing features in the Tmall dataset is greater than the improvement of introducing features in the MovleLens dataset. It may be because the MovieLens dataset is denser than the Tmall one, so the improvement brought by considering the features is not so obvious.

**Table 4:** Modeling sequence pattern from single sequence and multiple sequence.

**Dataset**	**Method**	**@5**		**@10**	
				**HR**	**NDCG**	**HR**	**NDCG**
Tmall	FIDS-item	0.3679	0.2679	0.4852	0.3057
		FIDS-fea	0.4271	0.3166	0.5423	0.3498
		FIDS	0.4361	0.3178	0.5493	0.3532
MovieLens	FIDS-item	0.6753	0.5082	0.7917	0.5462
		FIDS-fea	0.7432	0.5801	0.8361	0.6103
		FIDS	0.7469	0.5872	0.8376	0.6167

**(2) Whether feature interaction can positively help model performance**. To deeply explain the impact of feature interaction on FIDS, we remove the automatic feature interaction layer in the model, which is used to learning feature interaction and roughly integrate features to replace the module by averaging the features of each item (FIDS-mean) or using vanilla attention (FIDS-vani). Neither of these is designed to model integrated feature representations, and we can understand them only considering first-order features. Table 5 shows the experimental results.

**Table 5:** Impact of using feature averaging, vanilla attention, and feature interaction to integrate features.

**Dataset**	**Method**	**@5**		**@10**	
				**HR**	**NDCG**	**HR**	**NDCG**
Tmall	FIDS-mean	0.4079	0.2900	0.5405	0.3329
		FIDS-vani	0.3999	0.2914	0.5122	0.3309
		FIDS	0.4361	0.3178	0.5493	0.3532
MovieLens	FIDS-mean	0.7300	0.5589	0.8331	0.5925
		FIDS-vani	0.7315	0.5642	0.8285	0.5957
		FIDS	0.7469	0.5872	0.8376	0.6167

As expected, our model outperforms the other two models on both datasets. More specifically, the FIDS model is 6.06% and 3.00% better than FIDS-mean and 8.02% and 2.70% better than FIDS-vani on two datasets, respectively, which proves that learning the higher-order combined features can boost the performance of the model. In essence, the representation of features of each item is not independent and will be affected by other features. In our model, the self-attention networks in the automatic feature interaction layer establish the connection of different features. Features of an arbitrary order can also be connected through residual connections and stacking multiple self-attention blocks. In addition, we also observe that the experimental results of FIDS-vani and FIDS-mean are comparable. This shows that compared with the crude average operation, simply using vanilla attention to integrate features cannot improve the performance under the current two datasets.

## 6 Impact of hyper-parameters

In this section, we discuss the effect of hyper-parameters on the model. Due to space constraints, we only show results in terms of NDCG@10 on Tmall and MovieLens.

### 6.1 Impact of the residual connection

The essence of residual connection is to spread lower-layer information to higher-layer. In our model, the residual connection is also an indispensable part. There are a total of (*n*+2) self-attentions in the FIDS model, of which *n* self-attentions are used to learn feature interaction, and two self-attentions are used to model sequence patterns. To explore the role of residual connections in different tasks, we separately remove the residual connections in self-attention networks with different functions. We use Res-inter to represent the removal of the residual connections in the above *n* self-attentions, Res-seq to represent the removal of the residual connections in the above two self-attentions, and Res-seq-inter to remove all self-attention residual connections in FIDS. As shown in Table 6, in all evaluation metrics on the two datasets, the results of Res-seq exceed those of Res-seq-inter. This indicates that considering residual connections when learning feature interactions can indeed combine interactions of different orders. And Res-inter also performs better than Res-seq-inter on both datasets. This also shows that the residual connection helps propagate the visited items' embedding or integrated features' embedding to the following layer. Overall comparison, on the Tmall dataset, the performance of FIDS is improved by 4.39 % and 2.62% compared to that of Res-seq-inter in terms of HR and NDCG, respectively. On the MovieLens dataset, FIDS is improved by 1.34 % and 3.22 %, respectively. It shows that the residual connection promotes the performance of FIDS.

**Table 6:** Impact of the residual connection.

**Dataset**	**Method**	**@5**		**@10**	
				**HR**	**NDCG**	**HR**	**NDCG**
Tmall	Res-seq-inter	0.4122	0.3080	0.5334	0.3461
		Res-inter	0.4265	0.3108	0.5366	0.3463
		Res-seq	0.4243	0.3115	0.5437	0.3502
	FIDS	0.4361	0.3178	0.5493	0.3532
MovieLens	Res-seq-inter	0.7301	0.5664	0.8344	0.6001
		Res-inter	0.7404	0.5780	0.8364	0.6092
		Res-seq	0.7301	0.5676	0.8303	0.6003
	FIDS	0.7469	0.5872	0.8376	0.6167

### 6.2 Impact of the fully connected layer

Adding fully connected layers can endow the non-linear modeling capabilities of the self-attention module. To show this explicitly, we design to remove all fully connected layers in all self-attention networks. Table 7 shows the results where we use FIDS-fully to represent the model without the fully connected layer. On the Tmall dataset, FIDS has an average increase of 5.13% and 5.34% in terms of HR and NDCG, respectively, compared with FIDS-fully. On MovieLens, the percentages of improvement are 2.19% and 4.39% in terms of HR and NDCG, respectively. This shows that FIDS outperforms FIDS-fully and the learning ability of linear models is limited. Stacking fully connected layers endow FIDS with stronger learning ability.

**Table 7:** Impact of the fully connected layer.

**Dataset**	**Method**	**@5**		**@10**	
				**HR**	**NDCG**	**HR**	**NDCG**
Tmall	FIDS-fully	0.4097	0.2994	0.5291	0.3379
		FIDS	0.4361	0.3178	0.5493	0.3532
	MovieLens	FIDS-fully	0.7237	0.5597	0.8278	0.5937
		FIDS	0.7469	0.5872	0.8376	0.6167

### 6.3 Impact of the number of self-attention blocks

**Figure 2:**
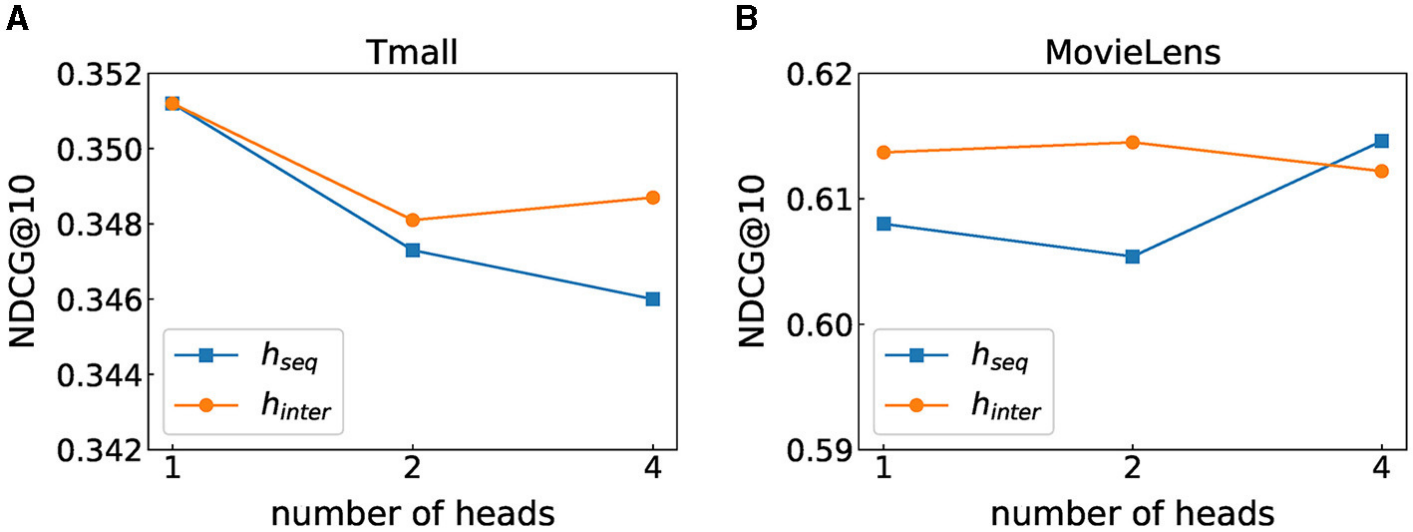
Performance under different number of heads. **(A)** Tmall dataset result. **(B)** MovieLens dataset result.

Stacking self-attention blocks on item sequences and feature sequences helps to learn more complex transition patterns, while higher-order feature interactions can be learned by stacking multiple blocks in the automatic feature interaction layer. The effect of the number of blocks on FIDS is shown in Figure 2, where *b*_*item*_, *b*_*inte*_, and *b*_*fea*_, respectively, denote the number of blocks in the item-wise self-attention layer, the feature-wise self-attention layer, and the automatic feature interaction layer. On both datasets, we can observe that setting the appropriate number of blocks can boost the performance of FIDS. However, when the number of blocks is too large, the performance is significantly reduced. Especially when *b*_*item*_ = 5, the result on MovieLens will quickly decrease to 0.2562 (we do not show in Figure 2). We analyze that it is easy to lose low-level information when too many blocks are stacked.

### 6.4 Impact of the number of self-attention heads

**Figure 3:**
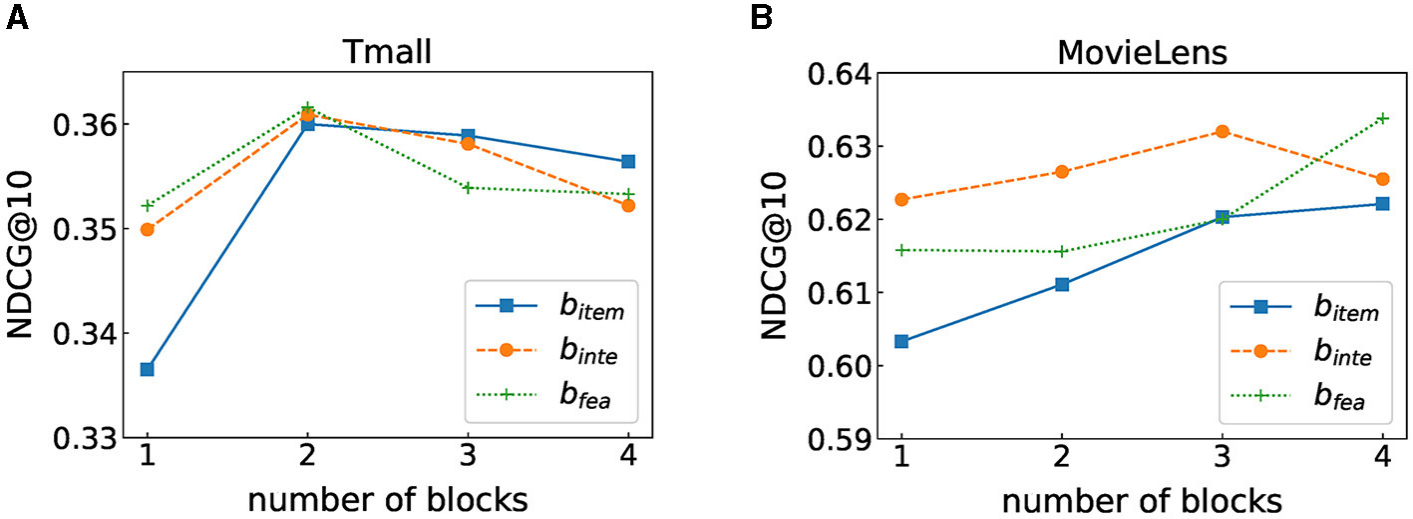
Performance under different number of blocks. **(A)** Tmall dataset result. **(B)** MovieLens dataset result.

Multi-head attention is to project Q, K, and V through multiple different linear transformations and finally stitch together different attention results, which intends to map features to different subspaces. We discuss the respective effects of multi-head in modeling sequence patterns and feature interactions. Figure 3 shows the experimental results, where *h*_*seq*_ represents the number of self-attention heads used for learning transition patterns and *h*_*inter*_ represents the number of self-attention heads learning feature interactions. We can observe that when the number of heads is 1, the model performs best on the Tmall dataset. On the MovieLens dataset, when *h*_*seq*_ = 4 and *h*_*inter*_ = 2, the value in terms of NDCG@10 is the largest. This may be because our model needs more heads to capture feature interactions and transition relationships as the MovieLens dataset contains more features, while there are fewer features involved in the Tmall dataset, and it may not require too complex structures to model these two relationships.

### 6.5 Impact of dropout rate

**Figure 4:**
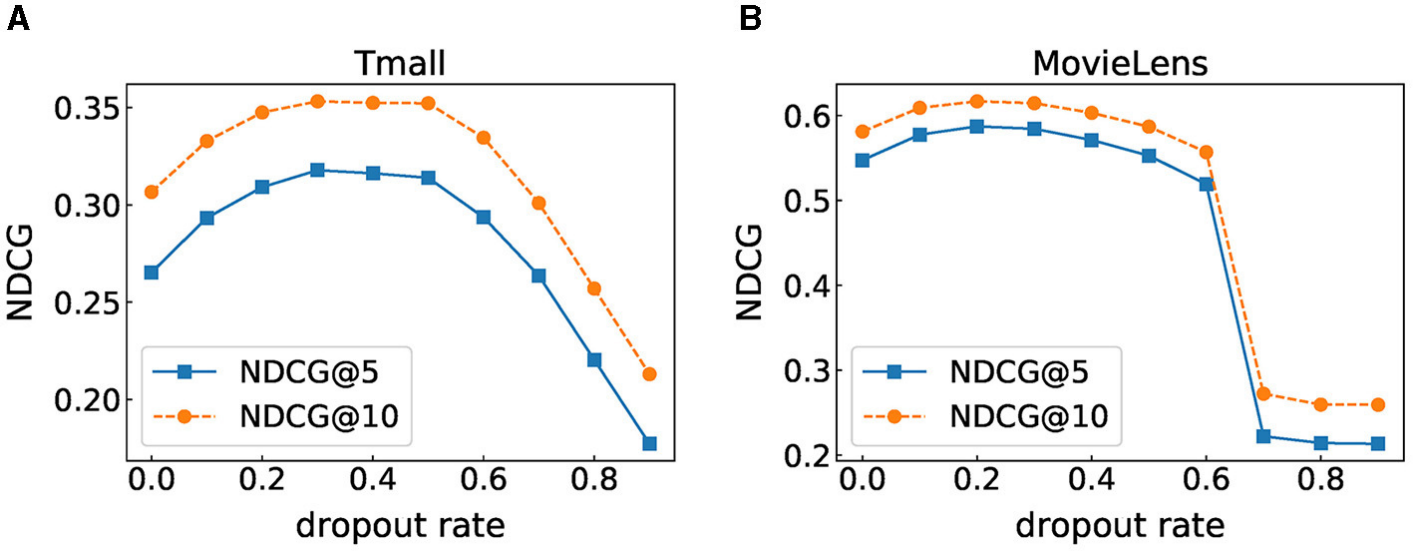
Performance under different dropout rate. **(A)** Tmall dataset result. **(B)** MovieLens dataset result.

Dropout is one of the effective means to solve overfitting. We also adopt dropout on the input item embedding, the fully connected layer, and the output of the “Mask”. To explore the impact of the dropout rate on model performance, we set the dropout rate to [0, 0.1, 0.2, ..., 0.8, 0.9] for experiments. Figure 4 shows the experimental results under different dropout rates. We can observe that when the dropout rate is 0.3, the experimental results on the Tmall dataset are the best, and the dropout rate of 0.2 is the most suitable for the MovieLens dataset. Setting the dropout rate to 0 means that no arbitrarily discarding information during training leads to poor results, which proves that the dropout strategy is indeed effective for overfitting. Moreover, both datasets show the same trend. As the dropout rate increases, the performance of the model first improves and then decreases or even drops sharply at the end, indicating that an appropriate dropout rate can improve the model's expressiveness and positively impact the generalization ability of FIDS. However, high dropout rates will inhibit the expression of the model.

### 6.6 Impact of the embedding size

**Figure 5:**
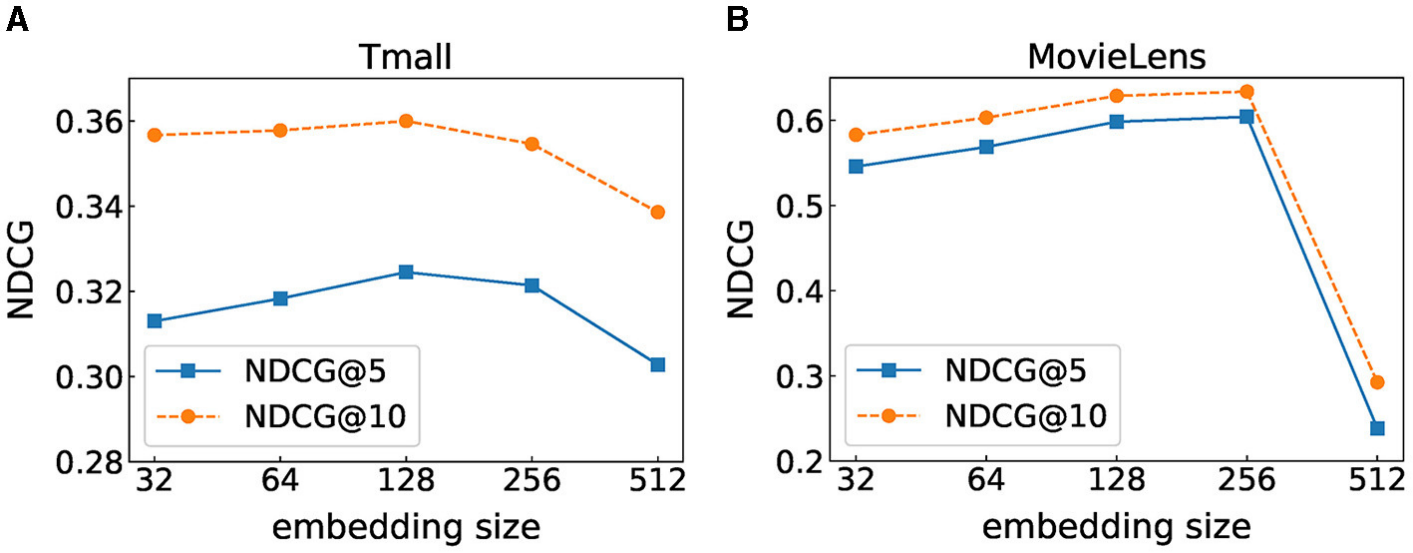
Performance under different embedding sizes. **(A)** Tmall dataset result. **(B)** MovieLens dataset result.

The embedding size is a crucial parameter that determines the accuracy of the recommendation. We set the embedding dimension in [64, 128, 256, 512] and show the performance of FIDS with different embedding sizes in terms of NDCG@5 and NDCG@10 in Figure 5. We can observe that setting the embedding size to 128 and 256 is the best choice for Tmall and MovieLens, respectively. The value of NDCG gradually increases as the embedding size increases until it reaches the highest point and then decreases as the embedding size increases. This is because FIDS can model more information on both datasets as the embedding size increases. However, overfitting may occur if the embedding size is too large. In addition, comparing the two datasets, the value of the optimal embedding size of MovieLens is greater than that of Tmall. We analyze that MovieLens contains denser data information, so a larger embedding size is needed to model the data.

## 7 Conclusion

In this study, we propose a novel model called Feature Interaction Dual Self-attention network (FIDS), which adopts dual self-attention to learn feature interactions and capture full sequential patterns. In particular, we apply multiple self-attention networks to capture feature interactions of each item to comprehensively and accurately represent the feature sequence. Then, we combine the effect of item sequence and feature sequence via full-connected layer for sequential recommendation. Extensive experimental analysis proves that our proposed model, FIDS, consistently exceeds the state-of-the-art methods, achieving an average improvement of 5.965% in HR and 4.66% in NDCG. Despite the promising results of the Feature Interaction Dual Self-attention network (FIDS), several future research directions can enhance its performance and applicability. Exploring advanced attention mechanisms, integrating with graph neural networks, and investigating dynamic feature representations can improve its ability to capture complex dependencies and interactions. Enhancing computational efficiency and scalability, developing real-time recommendation capabilities, and incorporating multi-modal data will broaden its applicability.

## Data Availability

The original contributions presented in the study are included in the article/supplementary material, further inquiries can be directed to the corresponding author.

## Footnotes

^1^ https://www.kaggle.com/vatsal73/movielens-1m

^2^ https://tianchi.aliyun.com/competition
